# Role of Surgical Therapy in Pulmonary Mucosa-Associated Lymphoid Tissue Lymphoma and Development of a Survival Nomogram

**DOI:** 10.1245/s10434-026-20313-z

**Published:** 2026-08-19

**Authors:** Ke Wang, Deyu Yang, Zhichao Li, Xinrong Chen, Tengjiao Lin, Linzhu Zhai

**Affiliations:** 1 Guangzhou University of Chinese Medicine, Guangzhou, China; 2 Lingnan Medical Research Center, Guangzhou University of Chinese Medicine, Guangzhou, China; 3 Cancer Center, Departments of Radiation Oncology, the First Affiliated Hospital of Guangzhou University of Chinese Medicine, Guangzhou, China

**Keywords:** Pulmonary MALT lymphoma, SEER, Treatment modality, Nomogram, Overall survival

## Abstract

**Background:**

This study aimed to investigate the clinical characteristics, prognostic factors, and treatment outcomes of patients with pulmonary mucosa-associated lymphoid tissue (MALT) lymphoma.

**Methods:**

The Surveillance, Epidemiology, and End Results (SEER) database was queried for patients with a diagnosis of pulmonary MALT lymphoma between 2000 and 2020.

**Results:**

Between 2000 and 2020, a total of 1721 patients had a diagnosis of pulmonary MALT lymphoma. Among the stages I and II patients, those treated with surgery outperformed those treated with radiotherapy in survival trends, but attention should be paid to potential selection bias. Among the patients who received combination therapy, those who received surgery combined with chemotherapy had longer overall survival (OS). Multifactorial Cox regression analysis identified sex, age, Ann Arbor stage, and surgery as prognostic factors for OS. A nomogram was established for pulmonary MALT lymphoma to predict OS, with a concordance index (C-index) of 0.718 and area under curve values of 0.7235, 0.7328, and 0.7917 for the 3-, 5-, and 10-year survival rates of the nomogram, respectively, indicating that the predicted probability of the calibration curve agreed well with the actual value.

**Conclusions:**

Pulmonary MALT lymphoma is a type of indolent lymphoma with a good prognosis, and surgery may be associated with improved patient survival outcomes, but this association needs to be further validated in prospective studies. This study established a nomogram that has good applicability for predicting long-term OS for patients.

**Supplementary Information:**

The online version contains supplementary material available at https://doi.org/10.1245/s10434-026-20313-z.

Primary pulmonary lymphoma is a lymphoma that occurs primarily in the lungs, accounting for 0.4% of all lymphomas and 0.5% to 1% of all primary lung tumors.^1,2^ The most common subtype of primary lung lymphoma is MALT lymphoma, accounting for 70% to 90% of cases,^3^ and it is considered an indolent lymphoma. The prevalence of pulmonary MALT lymphoma is increasing, with an incidence increase of 18% in 2006–2009 compared with 2001–2005.^4^

The etiology and pathogenesis of MALT lymphoma remain to be elucidated. Some studies have found that the disease is closely associated with autoimmune diseases and chronic infections (e.g., desiccation syndrome, systemic lupus erythematosus, and interstitial pneumonitis) and smoking.^5^

Unlike MALT lymphoma originating in the stomach, no infectious factors that play a role in the pathogenesis have been found in pulmonary MALT lymphoma.^6^ The incidence of pulmonary MALT lymphoma is low, and the clinical and imaging manifestations lack specificity. It is reported in the literature that 37.5 to 50.0% of patients are asymptomatic at the time of initial diagnosis, and that their disease is easily misdiagnosed as lung cancer or pneumonia.^7^

Due to the lack of large-scale, prospective clinical studies, treatment options for pulmonary MALT lymphoma are largely based on small-sample clinical studies and retrospective analyses. It is controversial which treatment modality, surgery or radiotherapy, is optimal for patients whose disease is diagnosed early. Currently, there are several possible treatments for pulmonary MALT lymphoma (watchful waiting, surgical removal, radiotherapy, or chemotherapy), which have been supplemented by the emergence of anti-CD20 monoclonal antibodies.^8–10^

A study by Lin et al.^11^ evaluated a cohort of 1742 patients with pulmonary MALT lymphoma using the SEER database, and their results showed that patients with stage I or II disease could benefit from surgery. In another analysis that included 953 cases of stages I and II pulmonary MALT lymphoma, the results showed that early-stage pulmonary MALT lymphoma is inherently indolent and that surgery did not improve OS.^12^ Importantly, the best treatment for stage I or II pulmonary MALT lymphoma, whether radiotherapy or surgical resection, has not been ascertained.^6^ These two near-contradictory studies may confuse clinicians’ decision-making about whether to accept surgical treatment for patients with stage I or II pulmonary MALT lymphoma or not. Therefore, further studies are required to assess the role of radiotherapy and surgical resection in the treatment of pulmonary MALT lymphoma.

The extensively used prognostic scoring system for MALT lymphoma considers age exceeding 70 years, elevated serum lactate dehydrogenase levels, and late stage as poor prognostic factors. The resulting MALT-International Prognostic Index score classifies MALT lymphoma into three risk subgroups: low, intermediate, and high.^13^ However, this prognostic scoring system does not include individual patient characteristics and treatment information, making it difficult to assess the impact of different individual characteristics and treatment modalities on survival. Therefore, development of a new, easily accessible prognostic model that includes individual characteristics and treatment information is required.

The incidence of pulmonary MALT lymphoma is low, and research data from large-scale prospective clinical trials are lacking. In contrast, the SEER database contains information on a large number of different cancer types in the United States population, which satisfactorily compensates for the inadequate number of cases in a single center. Therefore, we conducted a retrospective study based on the SEER database to summarize the clinical features and prognostic factors of pulmonary MALT lymphoma, analyzed the role of radiotherapy and surgical resection in the treatment of early-stage pulmonary MALT lymphoma, compared the effects of different combination treatment strategies, and established a nomogram to predict the prognosis for patients with pulmonary MALT lymphoma.

## Methods

### Data Sources

Patient information was obtained from the SEER database (version 8.4.2), which included patients with a first diagnosis of pulmonary MALT lymphoma from 2000 to 2020. The screening was performed using the International Classification of Diseases of Oncology, 3rd edition (ICD-O-3) histologic code 9699 and primary site codes C34.0, C34.1, C34.2, C34.3, C34.8, and C34.9. Unknown data on age at diagnosis, race, sex, marital status, and survival status were excluded. The data collected were demographic and clinical parameters including age, race, sex, marital status, tumor stage, treatment modality, and survival information. Clinical information on patients in the SEER database is publicly available and anonymous. Therefore, our study did not require ethics committee approval or patient consent.

### Statistical Analysis

Statistical analysis of the collected data was performed using R software (R version 4.2.3) to describe baseline information and clinical characteristics of the patient population. A multifactorial Cox regression analysis was used to screen the factors affecting patients’ prognoses.

Variable selection for multivariate Cox regression analysis primarily considers factors known to be clinically associated with OS such as age, tumor stage, and treatment modality, as well as confounding factors such as gender, race, and marital status. The hazard ratio (HR) and 95% confidence interval (CI) were used as the main indicators to measure the effect size. To control for potential confounding bias, we performed a 1:1 propensity score-matching (PSM) analysis of patients in the surgical and radiotherapy groups based on the characteristics of stages I and II patients. The Kaplan-Meier method was used to analyze the effects of radiotherapy and surgical treatment on OS for stages I and II patients and to compare the effects of different treatment combinations on OS.

We selected factors from the Cox regression analysis results that were significantly associated with OS and constructed a nomogram to forecast patients’ 3-, 5-, and 10-year OS rates. Subsequently, we divided the dataset into a training set and a validation set, and evaluated the accuracy and discrimination of this nomogram prediction by C-index, subject operating characteristic curve, and calibration curve. The C-index and area under curve (AUC) greater than 0.7 were considered to be sufficiently discriminative. A two-sided *P* value lower than 0.05 was considered statistically significant.

## Results

### Population Characteristics of Patients With Pulmonary MALT Lymphoma

**Fig. 1:**
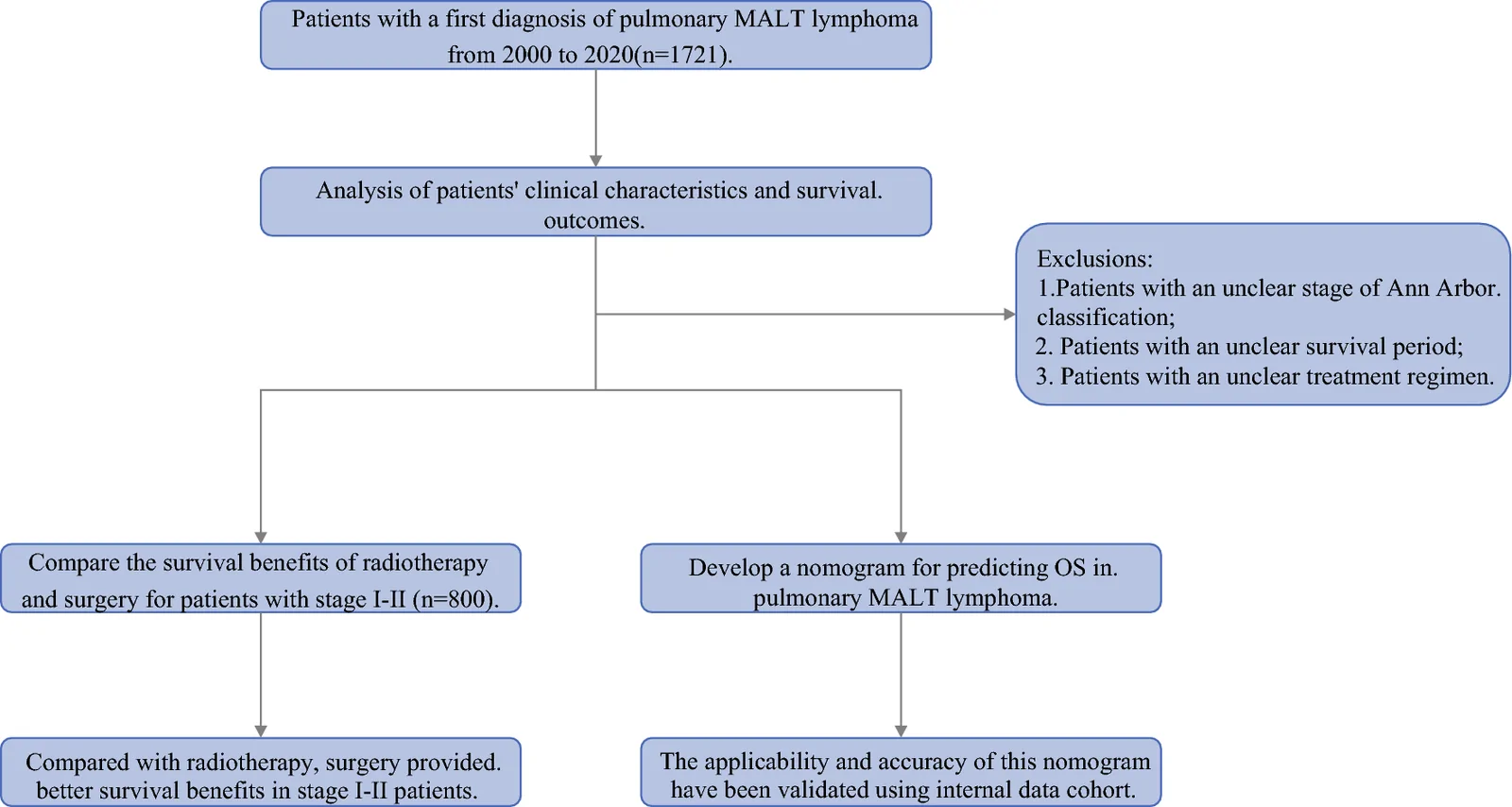
Incorporate the patient pathway flowchart

Patient characteristics are summarized in Table 1. The study included 1721 patients with a diagnosis of pulmonary MALT lymphoma in the United States between 2000 and 2020 (Fig. 1). Of all the patients, 724 (42.1%) were male and 997 (57.9%) were female, with a ratio of 1:1.37; 1467 (85.2%) were white; 143 (8.3%) were black; and 1,535 (89.2%) were married.

**Table 1:** Patient characteristics of pulmonary MALT lymphoma diagnosed in SEER, 2000–2020

Characteristic	No. of patients	Percentage (%)
Total	1721	100
*Age at diagnosis, years*		
Mean	67.54	
Median(range)	69(17-90)	
*Sex*		
Female	997	57.9
Male	724	42.1
*Race*		
White	1467	85.2
Black	143	8.3
Others^a^	111	6.4
*Marital status*		
Married^b^	1535	89.2
Single	186	10.8
*Ann arbor stage*		
Unknown	648	37.7
Stage I	664	38.6
Stage II	136	7.9
Stage III	23	1.3
Stage IV	250	14.5
*Surgery*		
No	1029	59.8
Yes	692	40.2
*Radiation*		
No	1536	89.3
Yes	185	10.7
*Chemotherapy*		
No	1235	71.8
Yes	486	28.2
*Cause of death (COD)*		
Alive	1082	62.9
Dead of other causes^c^	447	25.9
Disease-specific deaths	192	11.2

a American Indian/AK Native, Asian/Pacific Islander

b Included divorced/separated/widowed patients

c Included attributable to causes other than this cancer/missing

Clinical characteristics indicated that the Ann Arbor stage was unknown for 648 patients (37.7%), that 800 patients had (46.5%) stage I or II disease, and that 273 patients (15.8%) had stage III or IV disease. There were 692 (40.2%) patients who underwent surgery, 185 (10.7%) who received radiotherapy, and 486 (28.2%) who received chemotherapy. During the follow-up period, 639 (37.1%) deaths occurred, 192 (11.2%) of which were disease-specific.

**Fig. 2:**
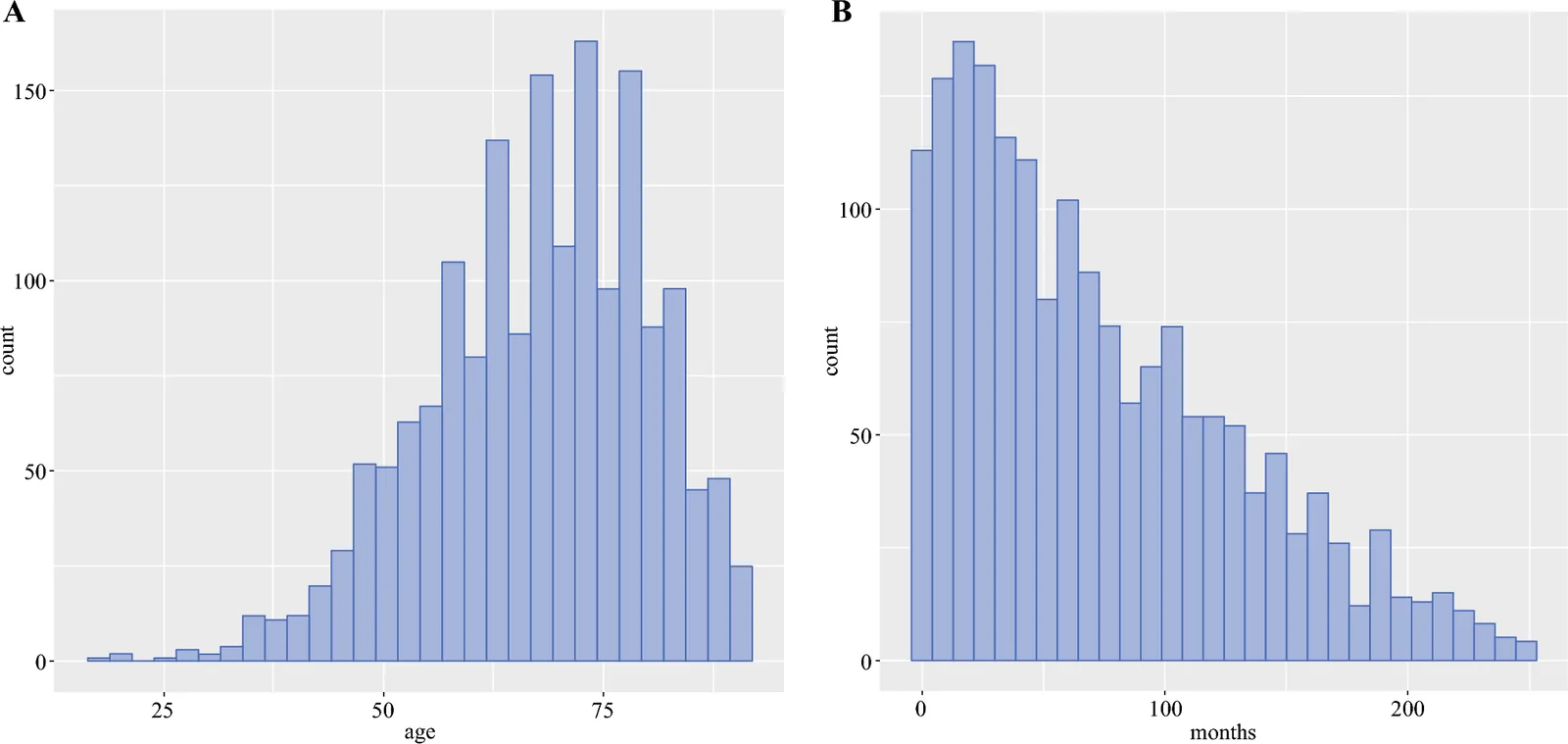
Distribution of age at diagnosis and survival time for the 2000–2020 cohort. (**A**) Histogram of the age distribution of the patients. The median age at diagnosis was 69 years. (**B**) Histogram of the survival time distribution of the patients. The median survival time was 59 months

The distribution of age at diagnosis and survival time for all the patients is presented in Fig. 2, with an age range of 17 to 90 years, a median age at diagnosis of 69 years, a median survival time of 59 months, and a mean survival time of 72.7 months.

### Multivariate Survival Analysis

A multivariate Cox regression analysis was performed. The results of prognostic factors influencing OS are shown in Table 2. Gender was significantly associated with OS. Male patients had a poorer prognosis than female patients (HR, 1.23; *P* = 0.02), and increasing age was significantly associated with an elevated risk of death (HR, 1.08; *P* < 0.001). Compared with patients who had stage I or II disease, those with stage III (HR, 1.88;* P* = 0.017) or stage IV (HR, 1.33; *P* = 0.008) disease had shorter OS. Surgical treatment tended to confer a better survival benefit (HR, 1.08; *P* < 0.001). However, factors such as race and marital status were not significantly associated with OS.

**Table 2:** Multivariate analysis of overall survival in patients with pulmonary MALT lymphoma

Covariate	HR^1^	95% CI^1^	*P*-value
*Sex*			
Female	Reference	–	
Male	1.23	1.03, 1.47	0.02
*Race*			
White	Reference	–	
Black	1.37	0.98, 1.93	0.067
Other	0.95	0.64,1.41	0.8
*Marital*			
Single	Reference	–	
Married	0.78	0.59, 1.03	0.084
Age	1.08	1.07, 1.09	< 0.001
*Surgery*			
No	Reference	–	
Yes	0.8	0.66, 0.97	0.024
*Radiation*			
No	Reference	–	
Yes	1	0.74, 1.35	> 0.9
*Chemo*			
No	Reference	–	
Yes	1.09	0.90, 1.33	0.4
*Ann arbor stage*			
Stage I	Reference	–	
Stage II	1.21	0.93, 1.58	0.2
Stage III	1.88	1.12, 3.17	0.017
Stage IV	1.33	1.08, 1.65	0.008

^1^HR = Hazard ratio, CI = Confidence interval

### Survival Outcomes

To compare survival outcomes between the surgical and radiotherapy cohorts for stages I and II patients, we excluded patients with unknown stage and included 458 patients with stage I or II disease and divided them into a surgery group and a radiotherapy group. Of these patients, 381 underwent surgery, and 162 died during the follow-up period, 77 received radiation therapy, and 41 died during the follow-up period.

**Fig. 3:**
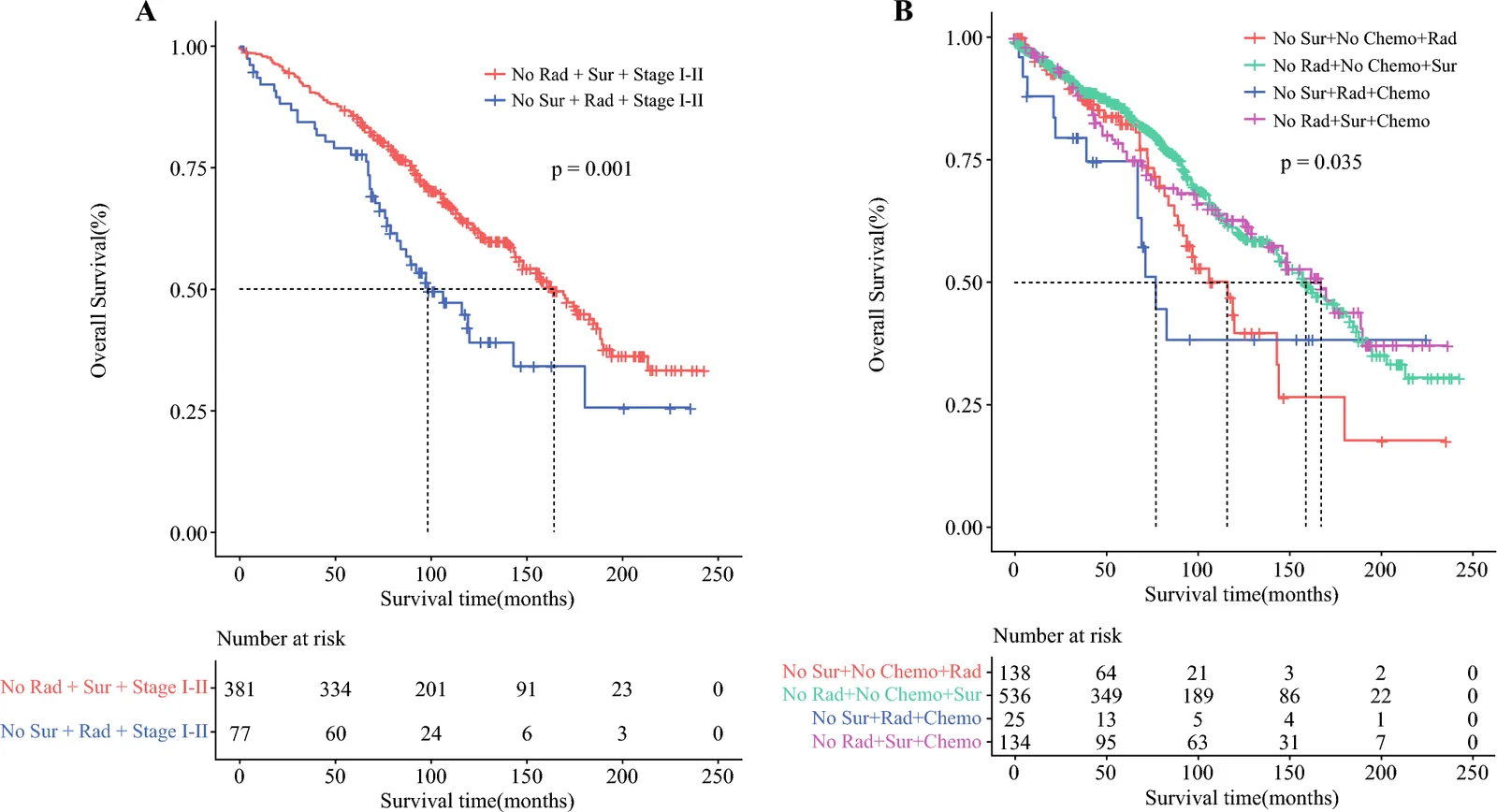
Kaplan–Meier survival curves stratified by treatment modalities. (**A**) Among stages I and II patients, overall survival (OS) was significantly worse for those who underwent radiotherapy than for those who received surgery (HR, 1.772; 95% CI, 1.255–2.504; *P* = 0.001). (**B**) Patients who received radiotherapy combined with chemotherapy had a worse prognosis than those who received surgery combined with chemotherapy (HR, 1.790; 95% CI, 1.437–3.342; *P* = 0.035)

As shown in Fig. 3A, among the stages I and II patients, OS was significantly worse for those who underwent radiotherapy than for those who received surgery (HR, 1.772; 95% CI, 1.255–2.504; *P* = 0.001). The median survival time for patients who underwent surgery was 164 months, with 3-, 5-, and 10-year survival rates of 91.6%, 85.8%, and 63.6%, respectively. The median survival time for patients who underwent radiotherapy was 98 months, with 3-, 5-, and 10-year survival rates of 84.3%, 77.7%, and 39.1%, respectively. The HR and 95% CI values for inter-group comparisons of Kaplan-Meier curves are shown in Table S1.

Based on baseline characteristics such as age, gender, race, marital status, and Ann Arbor stage, we performed a 1:1 PSM analysis comparing the surgical and radiotherapy groups of stages I and II patients. We found that 134 patients were successfully matched, with a good balance of measurable baseline variables between the two groups (Table S2). After PSM, the surgical group showed a trend toward higher OS than the radiotherapy group, with median survival of 157 months in the surgical group and 116 months in the radiotherapy group, (Fig. S1).

We included 833 patients who received different combination therapy regimens. During the follow-up period, 283 deaths occurred. We analyzed the impact of different combination therapy regimens on OS for patients receiving combination therapy. As shown in Fig. 3B, patients who received radiotherapy combined with chemotherapy had a worse prognosis than those who received surgery combined with chemotherapy (HR, 1.790; 95% CI, 1.437–3.342; *P* = 0.035). Among the 134 patients who received surgery combined with chemotherapy, the median survival time was 167 months, with 56 deaths during the follow-up period. Among the 25 patients who received radiotherapy combined with chemotherapy, the median survival time was 77 months, with 12 deaths during the follow-up period.

### Construction of the Nomogram

**Fig. 4:**
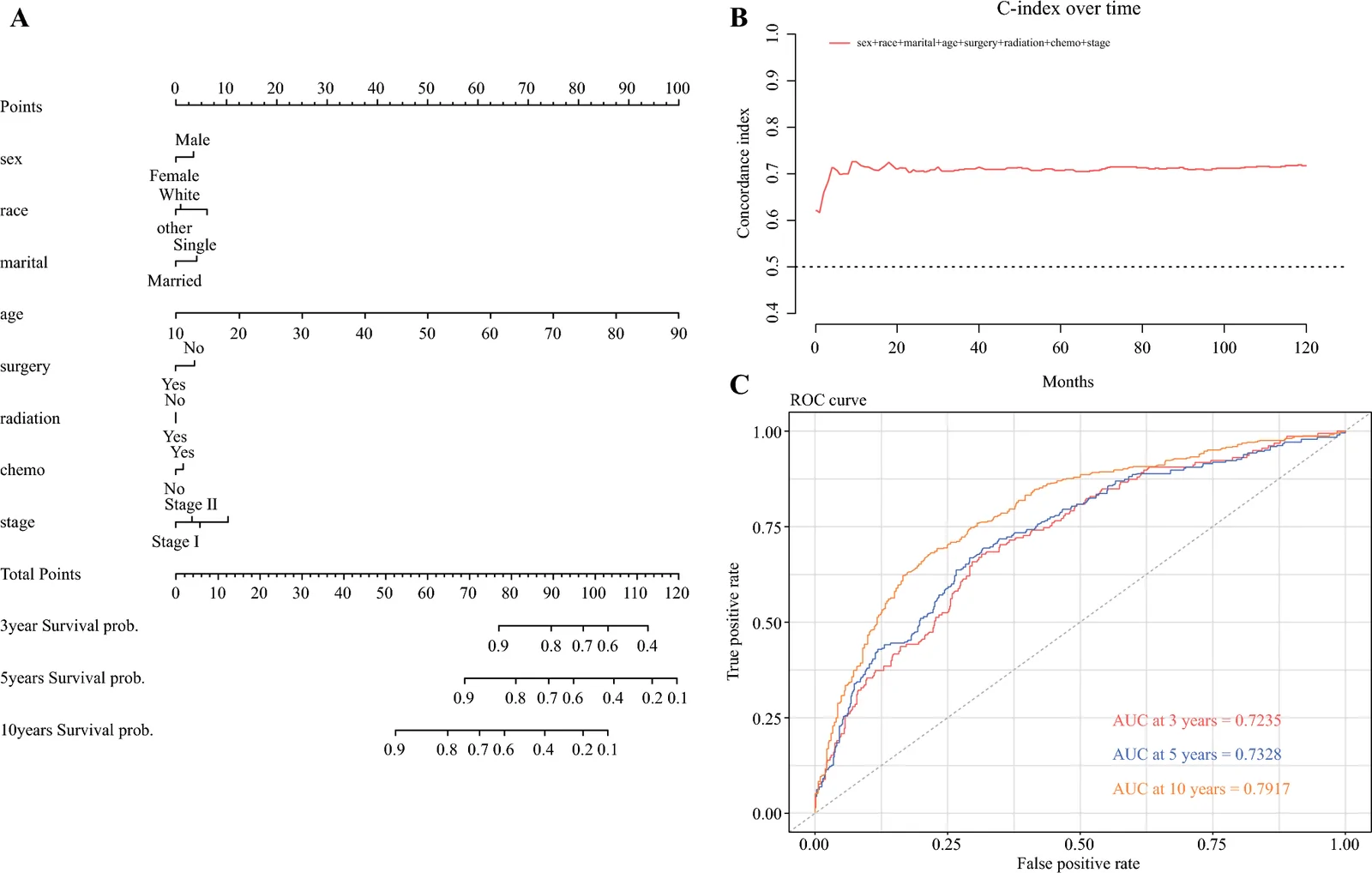
Survival nomogram, C-index, and ROC curves for patients with pulmonary MALT lymphoma. (**A**) Nomogram to predict 3-, 5-, and 10-year OS probability. The length of each bar reflects the variable’s contribution to the Cox model outcome. (**B**) C-index curves. **C** ROC curves for predicting patient survival at 3, 5, and 10 years. C-index, concordance index; ROC, receiver operating characteristic; AUC, area under the curve; MALT, mucosa-associated lymphoid tissue; OS, overall survival

Considering the lack of prognostic prediction models that include individual characteristics and treatment information of patients with pulmonary MALT lymphoma, after excluding 648 patients with unknown staging and their relevant information, we included clinical characteristics and treatment information of 1073 patients to establish a nomogram specifically for patients with pulmonary MALT lymphoma. As shown in Fig. 4, we developed a nomogram to predict patients’ 3-, 5-, and 10-year survival probabilities. The C-index of the nomogram was 0.718 (95% CI, 0.706–0.730), and the nomogram achieved time-dependent receiver operating characteristic–AUC values of 0.7235, 0.7328, and 0.7917 for the prediction of progression risks at 3, 5, and 10 years, respectively, indicating that the nomogram we constructed has a high discriminative ability. In addition, the accuracy of nomogram predictions was evaluated by calibration curves at 3, 5, and 10 years of survival, as presented in Fig. S2, with consistency between nomogram predictions and actual observations.

## Discussion

The most common pathologic subtype of marginal zone lymphoma (MZL) is MALT lymphoma, with the stomach as the primary site of origin and the lung as a relatively rare site of development. Compared with other pathologic subtypes of lymphoma, pulmonary MALT lymphoma is less aggressive and has a good prognosis.^14^

Given the low incidence of pulmonary MALT lymphoma, few large-sample retrospective studies of pulmonary MALT lymphoma have been conducted, and the findings are inconsistent. Therefore, we conducted a large-sample, population-based study that included 1721 patients with pulmonary MALT lymphoma. This study aimed to analyze the impact of surgical resection and radiotherapy on the prognosis of early-stage patients and to establish a prognostic nomogram containing individual patient characteristics and treatment information. The findings showed that among the included patients with pulmonary MALT lymphoma, the median age at diagnosis was 69 years, and the incidence of the disease was slightly higher among women than among men, with a ratio of 1.37:1. The highest prevalence was among white patients, with a proportion of 85.2%. The baseline characteristics of patients with the same trend have been mentioned in previous studies.^15,16^ During a follow-up period up to 20 years, only 192 patients died, with a disease-specific mortality rate of only 30% and a median survival time of 59 months for all patients, strongly suggesting that the survival prognosis of pulmonary MALT lymphoma, as a rare indolent lymphoma, is good.

During the follow-up period, 692 (40.2%) patients underwent surgical treatment, indicating that with the advancement of surgical techniques and the widespread use of thoracoscopic surgery, the treatment of surgical resection has become the choice of more patients. It is worth noting that although the Ann Arbor stage system is extensively used for the clinical stage of primary nodal lymphomas,^17^ for lymphomas outside the primary lymph nodes, such as primary pulmonary lymphoma, Ann Arbor stage is not always fully applicable, and there is some controversy about how to define stages III and IV patients in particular. In this study, the staging of 648 patients was unknown, accounting for 37.7% of the patients, which may have been related to the flaws in the staging system.

The treatment of pulmonary MALT lymphoma remains controversial, particularly stages I and II disease, and there is a lack of support from large prospective clinical studies concerning which treatment, surgical resection or radiotherapy, provides the greatest survival benefit. The results of a single-center retrospective study suggest that long-term progression-free survival can be achieved by surgical resection of limited pulmonary MALT lymphoma.^18^ This coincides with the findings of Lin et al.^12^^.^ However, it also has been found that watchful waiting is a better option for patients with stage I or II disease than surgery, which may be related to the fact that the study included patients in the timeframe of 1983 to 2015, which does not reflect the survival benefits of recent advances in surgical and radiation techniques.

Some studies have found that radiotherapy should be used as the first-line treatment for pulmonary MALT lymphoma to preserve lung function and reduce the risks associated with surgery.^19^ The study by Freret et al.^20^^.^ also concluded that radiotherapy has the advantage of being effective and safe in the management of pulmonary MALT lymphoma.

In our study, the survival time for the stages I and II patients treated with surgery was numerically higher than for the patients treated with radiotherapy, with a median survival ratio of 164 months versus 98 months. However this difference may have been influenced by unmeasured confounding factors such as physical fitness status, tumor burden, and surgical feasibility. We obtained the same trend in the analysis of combination treatment strategies, with a better prognosis for patients who received surgery combined with chemotherapy than for those who received radiotherapy combined with chemotherapy (median survival time ratio, 167 vs 77 months). Therefore, the results of this study indicate that surgical treatment is statistically associated with longer OS for patients with stage I or II pulmonary MALT lymphoma. The strategy of combining surgery with chemotherapy also shows a trend toward improved survival. These findings provide a reference for future clinical studies to explore the optimal treatment mode for early pulmonary MALT lymphoma.

It should be emphasized that due to the lack of descriptions of specific surgical procedures in the SEER database, it is not possible to determine the type of surgery performed for patients. Furthermore, patients who received radiation therapy may have been unable to undergo surgical resection due to surgical contraindications.

Given that the clinical presentation of pulmonary MALT lymphoma is atypical and imaging findings lack specificity, diagnosis relies primarily on pathologic examination, yet conventional biopsy is not feasible for patients with localized lesions. Therefore, surgical resection is of unique value in obtaining sufficient tissue specimens to clarify the pathologic diagnosis and to assess the scope of the disease, which is of reference significance for the development of follow-up treatment strategies.^21^ We also look forward to conducting prospective studies to compare the efficacy of surgery versus radiation therapy in the treatment of patients with stage I or II pulmonary MALT lymphoma.

The nomogram has become an effective tool for clinicians to use in visualization and rapid risk assessment for clinical decision-making.^22^ We incorporated patients’ individual characteristics and treatment information to construct a preliminary nomogram.

A composite score corresponding to sex, age, race, marital status, Ann Arbor stage, and treatment modality was calculated, which in turn predicted the patient’s probability of survival at 3, 5, and 10 years. This nomogram still needs external independent cohort validation. If validated in the future, it may assist clinicians in identifying high-risk patients and provide a reference for individualized follow-up strategies.

Different treatment modalities have different impacts on patients’ quality of life. A small number of patients are at higher risk for surgery due to their age and comorbidities, and they may die after surgery due to various complications. For early-stage patients, the risks and trauma of surgery are relatively small, and the treatment outcome is better. In the future, we plan to develop a user-friendly online calculator based on the predictive model established in this study. This tool will allow clinicians to enter patient variables in real time and immediately obtain corresponding survival predictions.

Our study had several additional limitations. First, the SEER database is a retrospective cohort, data from the United States, potential selection bias is unavoidable.

Second, many factors may affect a patient’s prognosis, but some specific data are not found in the SEER database (e.g., Karnofsky score, lesion size and number), and a degree of information bias due to the lack of this important clinical information may result in an inaccurate prediction of a patient’s prognosis. The SEER database did not record patients’ detailed surgical procedures, radiotherapy prescriptions, and chemotherapy regimens. Therefore, we were unable to conduct further comparisons between specific treatment options. Consequently, the association observed in this study between surgery and survival benefits in stages I and II patients cannot be directly interpreted as a causal relationship; it may be confounded by other factors.

Finally, the nomograms were established and verified using the same database, so it was necessary to verify the nomogram in another independent dataset. This may cause inaccuracies in the conclusions of this study. The results of this study should be interpreted with caution and require further validation through large-scale prospective studies or the collection of multicenter data that include a more comprehensive set of clinical characteristics. Nevertheless, our study provides important insights into the selection of treatment strategies and the prediction of survival time in pulmonary MALT lymphoma.

## Conclusion

In conclusion, a retrospective analysis of patients with pulmonary MALT lymphoma from 2000 to 2020 based on the SEER database showed that pulmonary MALT lymphoma, as a rare indolent lymphoma, is less aggressive and has a better overall prognosis. Stages I and II patients treated with surgery are associated with longer OS, and surgery combined with chemotherapy may be a combination therapeutic strategy worth exploring. Sex, age, Ann Arbor stage, and surgery are prognostic factors for OS. The nomogram constructed based on this still needs external validation. If validated in the future, it may provide a reference for clinicians to evaluate the long-term survival of patients.

## Supplementary Information

Below is the link to the electronic supplementary material.

Supplementary file1 (DOCX 320 kb)

## Data Availability

Publicly available datasets were analyzed in this study. These data can be found here: http://seer.cancer.gov.
