# Supplementary material for: Role of Surgical Therapy in Pulmonary Mucosa-Associated Lymphoid Tissue Lymphoma and Development of a Survival Nomogram
Source: Ann Surg Oncol. 2026 Aug 19;33(12):10366–74. doi: 10.1245/s10434-026-20313-z (PMC13675303; doi:10.1245/s10434-026-20313-z)
Supplement: Supplementary file 1 [file 10434_2026_20313_MOESM1_ESM.docx]

Supplementary Table 1. Hazard ratio and 95% confidence interval for intergroup comparison of KM curves in Figure 3

| **Intergroup Comparison** | **HR ^1^** | **95%CI ^2^** | ***P*-Value** |
| --- | --- | --- | --- |
| No Sur+Rad+StageI-II vs. No Rad + Sur +StageI-II | 1.772 | 1.255-2.504 | **0.001** |
| No Sur+No Chemo+Rad vs. No Rad + Sur + Chemo | 1.427 | 0.946-2.153 | 0.0903 |
| No Rad +No Chemo+ Sur vs. No Rad + Sur + Chemo | 0.963 | 0.712-1.302 | 0.807 |
| No Sur+ Rad + Chemo vs. No Rad + Sur + Chemo | 1.790 | 1.437-3.342 | **0.035** |

^1^ Hazard ratio

^2^ Confidence interval

Supplementary Table 2. Characteristics of patients in the surgery and radiotherapy

groups after PSM

| **Characteristic** | **Radiation Therapy** | **Surgery Group** | ***P* value** |
| --- | --- | --- | --- |
| **Age at diagnosis, years** |  |  | 0.157 |
| Mean | 69.85 | 67.01 |  |
| Median(range) | 71 | 68 |  |
| **Sex** |  |  |  |
| Female | 37 | 36 | 0.862 |
| Male | 30 | 31 |  |
| **Race** |  |  |  |
| White | 58 | 59 | 0.222 |
| Black | 6 | 2 |  |
| Others^3^ | 3 | 6 |  |
| **Marital status** |  |  | 0.129 |
| Married^4^ | 61 | 55 |  |
| Single | 6 | 12 |  |
| **Ann Arbor stage** |  |  | 0.116 |
| Stage I | 59 | 64 |  |
| Stage II | 8 | 3 |  |
| **Chemotherapy** |  |  | 1.000 |
| No | 55 | 55 |  |
| Yes | 12 | 12 |  |
|  |  |  |  |

^1^ American Indian/AK Native,Asian/Pacific Islander.

^2^ Included divorced/separated/widowed patients.

Supplementary Figure 1. Comparison of OS between surgery group and radiotherapy group in stage I-II patients after PSM analysis.

Supplementary Figure 2. Calibration curve of the nomogram for the prediction of 3-, 5-, and 10-year OS (A–C). The abscissa represents the nomogram-predicted survival rate, and the ordinate represents the actual survival rate. The calibration curves for 3-, 5-, and 10-year survival rates exhibited satisfactory agreements between the predicted and actual values. OS, overall survival.
